# Case of Subcutaneous Emphysema Occurring in the Course of Pneumonia

**Published:** 1854-01

**Authors:** J. Forsyth Meigs


### *Case of Subcutaneous Emphysema occurring in the course of Pneumonia.* By J. Forsyth Meigs, M. D.

The subject of this case, a boy between six and seven years of age, had an attack of pneumonia of the upper third of the right lung in April, 1849. From that attack, which occurred in this city, under my charge, he recovered entirely, and remained well until July of the same year, when he was seized with dysentery in a town at some distance from this place. The dysentery was violent, and continued so until the 21st day, when he began to mend. On the 24th and 25th days he continued better, so as to sit up a little each day. On the 25th day, the mother observed that he coughed a little. On the 26th, he had a warm bath, and on the 27th and 28th days he had more cough, which was short and teasing, and he seemed more sick and drooping than before, and had some wheezing in his breathing. During the night of the 29th day, he was very restless, complained of choking and smothering, and of weight at the top of the sternum and about the neck, and coughed a great deal. On the morning of the 30th day, the mother discovered, while in the act of washing him, a swelling or puffing of the right side of the neck, extending also towards the front of the neck and top of the sternum. Later in the day, after having gone down stairs, she was astonished to find on returning, a soft swelling as large as a small black walnut, over the front edge of the right masseter muscle. His dysentery had now subsided into a feculent diarrhœa.

On the next day but one after this, August 8th, 1849, he was brought to town and placed under my charge. This was the 32d day since he was seized with the dysentery, and the 8th since he began to cough.

*August 8th. Afternoon.* The child looks most wretchedly. He is very much emaciated, being merely skin and bone. His surface is of a dirty pale color. He is very weak, whining, fretful and irritable. He coughs very much, rather loosely, and without apparent pain. There is no expectoration. Respiration rather frequent. Auscultation detects bronchial respiration over the summit of the left lung behind, while in the same region there is dulness on percussion. Elsewhere the physical signs are healthy. The left side of the neck, from the ear downwards to about four inches below the clavicle; from the lateral edge of the nucha outwards to the acromion and inwards to two inches to the right of the mesial line of the neck, and the parts about the top of the sternum for several inches, are all tumefied so as to project very considerably beyond the surrounding surfaces, presenting an appearance as though an irregular cushion had been placed upon the skin. The swelling is soft, without fluctuation, giving precisely the impression of air, though, at the time, I did not note the existence of crepitation. The integument over the swelling is a little reddish and inflamed, and somewhat tender to pressure. Over the anterior edge of the right masseter muscle is a distinctly circumscribed and projecting swelling, looking exactly as though the child were holding in his cheek a large hickory-nut. This, like the other, is elastic, soft, and without fluctuation.

The patient was put upon a diet of milk punch, soup, bread, and small quantities of light meats. One grain of quinine with one drop of solution of the iodide of iron, were ordered to be given every three hours. An injection of twenty drops of laudanum in starch water was to be administered at night, and ten drops of solution of morphia with ten of sweet spirits of nitre were to be given internally, two or three times in the course of the night, if necessary.

*August 9th.* No decided change, except that the appetite is improved. In the evening the swelling had extended down the left arm to the wrist. It was not very perceptible to the eye, but the touch detected the soft, puffy feeling, caused by the extravasated air ; and now the peculiar crepitation of emphysema was very marked. Treatment continued.

*August 10th.* Has had a quiet night, and looks rather better, though very languid and very thin. He coughs much more, and expectorates a good deal of mucus tinged with scarlet blood. He is very irritable. The pulse is 120, and of moderate force ; the skin is of the natural temperature, but dryish. The tongue is clean and moist. Auscultation reveals crepitant râle with bronchial respiration over the upper third of the left lung behind. Over the same region the percussion is very dull. The bowels have been open six times in the last twenty-four hours ; the stools consist of semi-fluid feculent matter, without blood or mucus. The swelling about the neck and left shoulder has diffused itself more equally, so that the tumefaction has apparently diminished. It is more evident on the back and down the left arm. At the same time, it is less painful to the touch than before, and the integument over the swelling is less red. Treatment continued.

In the evening the pulse was 112, and the respiration 24.

*August 18th.* Up to this time the child improved gradually, though very slowly. The cough became looser, less frequent, and easier. The expectoration, moderately free at first, has diminished progressively, and consists now of whitish and frothy mucus, without any appearance of blood. The respiration has been easy, and never over 30. The crepitation perceived upon auscultation has diminished in abundance, while the bronchial respiration and dulness on percussion have continued much the same. The pulse has seldom been over 112. There has been a slight increase of feverishness towards night, marked by dryness and harshness of the skin. The appetite has been pretty good, though only for certain articles. The bowels were moved from four to six times a day, and the discharges were natural in color and odor. The temper has been very irritable and difficult, and there has been a good deal of wandering, with muttering delirium, though the child was always intelligent when aroused.

The emphysema extended gradually until it could be detected over both temples above the ears, and all over the occipital region of the head. It was perceptible all round the neck, down the left arm to the back of the hand, over the whole dorsum of the body down to the coccyx, but not in the legs, over the front of the thorax, and over the abdomen down to the crista of the ilia. It did not extend to the right arm, nor did it affect the lower extremities at all. While being diffused in this way, the large prominence in front and on the left side of the neck subsided little by little, until these parts returned nearly to their natural size; at the same time, the globular swelling on the right masseter muscle also diminished very much.

The treatment above mentioned was continued without change. Nothing special was done for the emphysema.

*August 30th.* Since the last visit the patient has improved very much. He is gaining flesh, walks about the room, and is dressed all day. The expression is natural and the temper cheerful. The appetite is excellent and the bowels natural. The cough is nearly gone, and there is no expectoration. The pulse and respiration are natural. The emphysema has gradually disappeared, until, three days ago, there was none of it to be found.

The treatment has not been changed. He has taken four doses of the quinine and iodide of iron daily, and a little wine and water.

*September 1st.* Auscultation and percussion practised carefully to-day reveal nothing abnormal.

Treatment suspended, except that half a grain of metallic iron is to be taken three times a day, until he shall have regained his natural strength and color.

*Remarks.* There can be no doubt that, in the above case, the external emphysema was the result of an extravasation of air into the cellular tissue of the lungs, by rupture during an effort of coughing, of some of the air-vesicles. From the point or points of the lung-tissue into which the air was extravasated, it made its way to the root of the lungs, and thence through the mediastinum to the cellular tissue of the lower part of the neck, of the face, chest, upper extremities, and trunk of the body. After coughing very much for three days, the patient complained during the night of choking and smothering, and of weight at the top of the sternum and about the neck, and on the following morning the mother discovered the swelling and puffing at the lower right side of the neck and upper part of the sternum, which proved to be the beginning of an extensive subcutaneous emphysema.

The mode of development of the emphysema in this case, was the same, of course, as in that which has been observed not unfrequently to follow violent muscular efforts, as in lifting heavy weights and in the throes of child-birth; and in that also which has been produced by an obstacle to the act of respiration, as in hooping cough, bronchitis, hysteria, and in that dependent on the presence of foreign bodies in the upper air passages. The examples of emphysema caused in these modes are now too numerous and well authenticated to allow of any other explanation than the above, and especially of the one made use of by some of the older authors, who supposed the air to have been thrown out into the cellular tissue by an act of secretion or decomposition.

